# Production of Agglomerates, Composite Materials, and Seed Coatings from Tannery Waste as New Methods for Its Management

**DOI:** 10.3390/ma14216695

**Published:** 2021-11-06

**Authors:** Katarzyna Ławińska

**Affiliations:** Łukasiewicz Research Network—Institute of Leather Industry, Zgierska 73, 91-463 Lodz, Poland; katarzyna.lawinska@ips.lukasiewicz.gov.pl

**Keywords:** tanning shavings, waste collagen, composite, granulation, biostimulants

## Abstract

This paper presents methods for managing waste produced by the leather industry, including tanning shavings derived from chrome tanning technologies and collagen preparations. Shavings were classified according to their shape (in accordance with Zingg’s shape classification). The content of individual elements was determined, together with the content of volatile organic compounds. Two new products were developed as part of the completed works: agglomerates (methods of non-pressure granulation) and composite materials were produced on the basis of tanning shavings and mineral fillers. Young’s modulus values classify these composite materials in the group of polymers and certain materials from the group of elastomers. A method for seed coating (on the example of legumes and rape) was also developed using a disc granulator, including collagen preparations in one of the layers as a solution for preventing the effects of droughts (biostimulant). The analyses of selected properties of the new products confirm the wide possible application of waste shavings and collagen preparations in a circular economy, especially in the construction, packaging, and agricultural sectors.

## 1. Introduction

Leather is the tanning sector’s main output and an intermediate industrial product, with applications in downstream sectors of the consumer goods industry (such as footwear, clothes, furniture, and the automotive sector). Waste generated by the leather industry, due to its composition, may be used as a raw material for manufacturing new economic products, thus ensuring sustainable and eco-friendly industrial practices. Solid leather waste can be considered a composite material that is highly structured and connected with collagen fibers [1]. Different forms of solid waste are produced during rawhide processing, including tannery fleshing waste (TFW) (viscous and biodegradable products including 8% salt, 8% collagen, 4% fats, and 80% water [2]), chrome shavings, chrome splits and buffing dust, skin trimmings, and hair [3]. In order to efficiently manage waste, the TFW manufacturing residue has been used, for example, for biogas [4], biodiesel [2,5,6], or biofuels and economic biochar [7,8] with potential application as a soil biofertilizer or as a low-cost bioabsorbent. The authors of papers [9,10,11] pointed to the use of leather shavings as alternative adsorbents, e.g., for the treatment of wastewater containing dyes.

In adsorbent materials, in order to remove volatile organic compound (VOC) pollutants, microporous activated carbons [12,13,14,15,16] were obtained from vegetable-tanned leather waste (shavings, buffing dust) [17]. In study [18], the ability of chrome shavings to remove motor oils, oily wastes, and hydrocarbons from water was studied. Solid residue pyrolysis can be adopted as an innovative solution by tanners and leather producers. Thermal decomposition of leather waste begins at 80 °C, and its maximum is 325 °C [19]. Paper [20] provided a strategy for the high-value use of chrome shavings (by pyrolyzing) and a practical method for the removal of antibiotics. Study [21] provided basic data for the determination of pyrolysis temperatures of chrome-tanned leather shavings targeting different products. Moreover, the authors of papers [22,23,24,25,26] developed a model for simulating and predicting the pyrolysis behavior of chrome shavings. A pretreatment can increase the feasibility of producing energy with chromium leather waste [27] and can reduce the costs of energy consumption, waste disposal, eliminate solid waste, and reduce greenhouse gas emissions from microbiological degradation of tannery waste [28,29]. The removal and recovery of heavy metals from tannery sludge subjected to a plasma pyro-gasification process was described in [30]. The gasification of biochar derived from tanning shavings resulted in two main products: a hydrogen-rich syngas, which has applications such as electricity generation and chemical production, and a chromium-rich ash, which can potentially be utilized, e.g., in stainless-steel manufacturing processes [31]. Olejnik, in his work [32,33,34], focused on the different aspects of process efficiency and reducing the technology impact on the environment, with reference to economy and pollutant interaction.

Chromium is a valuable resource, and its reuse is economical and environmentally feasible [35]. Chrome is the most successful tanning agent used in leather tanning processes (due to its ability to produce leather with attributes desirable for high-quality leather: hydrothermal stability, better dyeing characteristics and softness). Chromium recovered from chrome-tanned leather shavings showed the possibility of producing pigments for paints and paint coats [36]. Protein from chrome shavings may be reused after chemical modification as a replacement for commercial retanning products [37]. Moreover, the collagen hydrolysate obtained from chromed shavings after alkali and enzymatic hydrolysis is suitable as a component of leather finishing coatings (as a 5% addition) [38]. In study [39], a protein-based product was developed from raw trimming waste and used in a chrome tanning process to enhance the exhaustion level of chromium.

Paper [40] highlights the emerging body of knowledge and research on chromium minimization, recycling and/or reuse of chromium waste to make tanneries safer for people and more eco-friendly. A scheme for the total control of chromium in the leather industry was designed in paper [41]. Long et al. [42] described a method for recovering Cr (VI) from tannery sludge and chrome-tanned leather shavings. There is also tanning technology based on wet white and a combination method, which involves the mixing of tanning agents [43,44]. Leather waste is disposed of in landfills; however, the composting of leather with food waste is a promising alternative to landfills [45].

In line with environmental awareness, the idea of a circular economy, and an increased interest in eco-friendly technologies, the objective of this study is to present methods explored by the Łukasiewicz Research Network, Institute of Leather Industry, to minimize the production and management of waste from the leather industry. So far, natural leather is still the best material for various products, especially footwear. The presented actions in terms of processing selected waste from the leather industry are consistent with the European Green Deal, which is a set of policy initiatives aimed at securing the sustainable economy of the EU.

## 2. Materials and Methods

This paper describes the use of unit processes in methods of processing waste generated by the leather industry on the basis of chromium tanning shavings and waste collagen preparations for the purpose of their reuse. The granulometric composition of shavings was determined on the basis of screen analysis [46,47], taking into account sieve hole blocking [48,49,50,51]. Zingg’s classification was used for analyzing the shavings shape (using a KAMIKA Instruments analyzer, Warsa, Poland). On the basis of this classification, four basic shapes of grains were differentiated: disc-shaped, spherical, bladed, and rod-like. The method of a falling grain scanning was used in a three-dimensional measurement (two dimensions were obtained on the basis of optical measurements of transducers, while the third dimension was determined based on how many times each grain was scanned). The chemical composition of tanning shavings samples in terms of their content of selected elements (metals) was determined using inductively coupled plasma-optical emission spectrometry (ICP-OES) [52]. In these tests, shavings samples (0.2 g) were placed in a Teflon vessel, and 6 cm^3^ of HNO_3_ (65%) were added. Next, they were mineralized using the Magnum II (ERTEC-POLAND Edward Reszke, Wroclaw, Poland) microwave reactor in 3 cycles totaling 20 min, at 300 °C and at pressure rising to 45 bar. Clear digest solutions were quantitatively transferred to 25 cm^3^ flasks, and demineralized water was added. All of the tested samples were analyzed using the inductively coupled plasma-atomic emission spectrometry with the ICP-OES 5110 spectrometer (Agilent, Santa Clara, CA, USA). The content of the tested elements in the shavings samples was read using calibration curves developed on the basis of the individual metal models.

The content of volatile organic compounds (VOC) was determined using the gas chromatography method with a mass detector (GC/MS/HS) at 150 °C. The preparation method involved the direct heating of a sample placed in a vial (sample mass of 0.2 g) in the chromatograph furnace; then, the emitted compounds were chromatographically separated. Samples were analyzed using the 2030 Nexis GC (Shimadzu Corporation, Kyoto, Japan) chromatograph fitted with MS GCMS-QP2020 (Shimadzu Corporation, Kyoto, Japan) and a headspace-type AOC-20i autosampler (Shimadzu Corporation, Kyoto, Japan). A chromatography column dedicated for the separation and quantification of VOC, ZB-624plus (Phenomenex Companies Worldwide, Torrance, CA, USA) was used. The furnace temperature was initially maintained at 40 °C for 1 min and then increased by 10 °C∙min^−1^ to 150 °C. The injection volume was 1.0 cm^3^, while the separation ratio was 1:10. The model VOC solution (mixture) was purchased from Sigma-Aldrich (Taufkirchen, Germany).

As part of the research, new composite materials were produced, containing 60% characterized tanning shavings and 40% adhesive medium (four types were used: a homopolymer that is a water dispersion of polyvinyl acetate, modified animal-derived gelatin glue, low-ammonia natural latex glue, and solvent-free colorless epoxy resin with a hardening agent) and a mineral filler: dolomite, kaolin, or bentonite (mineral content of 10% in relation to the shavings’ mass) [53,54]. The mixture of all components was pressed in a hydraulic press at a constant pressure of 20 MPa with the possibility of heating. The produced 280 mm × 280 mm composites were dried in a laboratory drier at 25 °C for 24 h.

The produced composite materials were subjected to static tensile tests using a Zwick/Roell Z010 type testing machine (Zwick Roell Group, Ulm, Germany). Furthermore, for individual composite materials, their capacity for water absorption (absorbability) and for water release during the drying process was determined [50]. In view of the environmental aspect, analyses of the extracted chromium VI content (using the spectrophotometric method with 1,5-diphenylcarbazide) were performed.

Disc granulation [55,56,57] was selected for processing the shavings. The selected method made it possible to produce particles with a proper shape, dimensions, and physical and chemical properties. In addition, it is also a relatively easy to use, cost-efficient, and environmentally-friendly (waste-free) method that is effective for fine-grained materials. Wet materials may also be granulated using this method.

Non-pressure granulation methods were developed in order to give the shavings regular, spherical shapes and produce a loose regular deposit. A disc granulator (Lodz University of Technology, Lodz, Poland) [58,59] and a vibrating disc granulator (Lodz University of Technology, Lodz, Poland) [60] were used for shavings granulation. Granulation was run until the majority of the fine-grained material was attached to the granules, ensuring that the agglomeration of granules did not occur (no coalescence phenomenon).

The following methods of producing granulated products were developed and tested. In the first method, a mixture of shavings and a fine-grained mineral material was granulated. Shavings were poured onto the granulator disc, and then, a dry mineral component was added in the form of powdered dolomite or gypsum. Granulation was carried out with simultaneous deposit drenching with a 50 or 75% soluble glass solution, using a hydraulic nozzle, in the form of drops of 0.01–0.5 mm in diameter [59]. The second method involved the soaking of the shavings with the binding liquid beforehand (shavings previously mixed with a water glass solution) and then granulating the wet pulp with the addition of a selected fine-grained mineral material (dolomite or gypsum). A 50% aqueous solution of water glass was used in the tests. The wet pulp was granulated on a rotating disc [60]. In the third method, a vibrating disc granulator was used. The addition of a 75% solution of sodium water glass (in the wet pulp), dolomite, and gypsum was applied. No binding liquid was added in the granulation process [60].

Waste collagen preparations were used as a component in the processes of producing seed coatings for the purpose of increasing the seeds’ resistance to drought through improving moisture conditions at the stage of seed germination. The characteristics of the selected preparations are given in paper [61]. Disc granulation was also used to form coatings for the seeds of legumes (peas, broad bean, and soybean) and rape, on the basis of waste collagen preparations derived from alkaline hydrolysis [62,63]. The coating processes involved the use of natural additives facilitating germination, such as dolomite, chalk, and kaolin, as well as peat and soot. Furthermore, the environmental aspect (circular economy) was taken into account when selecting different types of the binding liquid; i.e., solutions of molasses (a waste product from the sugar industry) were used. The individual layers of the coating were formed in a specific order. The centrally placed seed was first coated with a layer of collagen hydrolysate or a solution of yellow dextrin and polyvinyl alcohol or a solution of molasses. The outer layer was a mineral additive. The impact of the coating composition on the initial growth of the plants was determined, above all, through the measurement of seedlings length. Growth was observed for 7 and 10 days.

## 3. Results

### 3.1. Characteristics of Tanning Shavings

**Figure 1:**
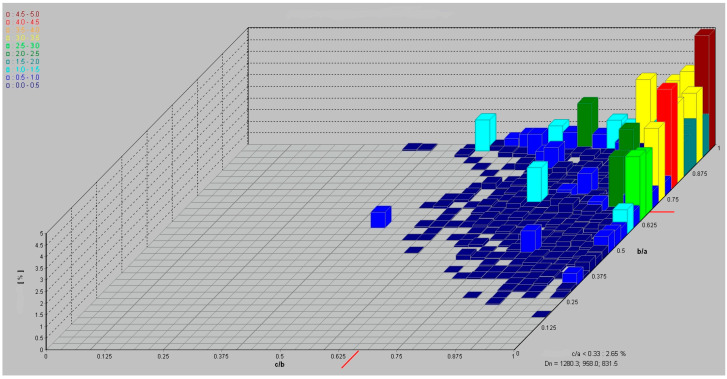
Shapes of shavings (in accordance with Zingg’s classification).

The executed processes of tanning shavings screening showed that over 80% of their composition constituted grains of less than 8 mm, with approximately 35% of those being below 1 mm. Dry tanning shavings were characterized by very low bulk density that did not exceed 0.1 g/cm^3^. The pore structure of the tested tanning shaving samples consisted mainly of mesopores and macropores (a relatively low BET (Brunauer–Emmett–Teller) specific surface area (SSA) in the range of 2.55–3.51 m^2^/g) [60]. The results of the shavings shape analysis are given in Table 1. The dominant shapes of shavings were spherical (70.14%) and rod-like (23.77%) (in accordance with Zingg’s classification, Figure 1).

The results of the analysis of the tanning shaving samples’ chemical composition are given in Table 2. The performed analyses showed that the tested waste contained significant amounts of elements. Tanning shavings can be considered useful waste in view of their elemental composition, due to a high content of Ca, Mg, S, and P as a valuable source of macroelements and structural elements. The high content of Ca, as well as Na in shavings, is also mentioned by the authors of paper [62]. The lack of, above all, Pb and As is important in view of the environmental aspect of the discussion.

The organic acids, preservatives, surfactants, and alkanes obtained as a result of the VOC analysis were the components of chemical preparations used during leather expedition and tanning (Table 3). Substances (e.g., hexadecanal) that are, according to the CLP Regulation (Regulation (Ec) No. 1272/2008 of the European Parliament and of the Council of 16 December 2008 on the classification, labelling, and packaging of substances and mixtures, amending and repealing Directives 67/548/EEC and 1999/45/EC, and amending Regulation (EC) No 1907/2006), classified as irritating or harmful to the environment, represented a small percentage of the analyzed waste.

### 3.2. Composite Material Made of Waste Tanning Shavings and Mineral Fillers

During the tests, new composite materials were produced based on fragmented collagen fibers contained in tanning shavings, derived from the processing of the wet blue leather semi-finished product. The described tests and their results prove that it is possible to produce composite materials based on waste tanning shavings and widely available mineral fillers and use them again in a closed cycle. As a result of the research, the optimal composition of a composite material made of collagen fibers derived from the leather industry waste and mineral fillers was determined.

When analyzing the values of the Young’s moduli (Table 4) obtained for the produced composite materials, they can be classified within polymer foam (VLD, LD, and MD) and some materials from the elastomer group (IR, CR, and EVA), i.e., materials capable of reversible deformations under the impact of mechanical forces without the risk of losing the continuity of their structure, which significantly expands the potential areas of their application [48]. Summarizing [53,54], the type of the adhesive medium significantly impacts the ability to absorb and release water. Depending on the intended purpose of a given material, its ability to absorb water may be reduced or increased, as can its drying rate. In relation to environmental tests, composite materials made of collagen fibers derived from the leather industry waste and mineral fillers are safe to use, which is proven by the lack of Cr (VI) content.

### 3.3. Obtaining Granules from Waste Tanning Shavings

The developed methods of processing shavings make it possible to produce agglomerates with proper dimensions, a shape close to a sphere, and relatively high mechanical strength. It is also possible to use other waste materials, such as for example fine-grained gypsum, which together with a drying process ensures granules that are dry on the outside, that form a non-lumping deposit loose enough for the produced agglomerates to be transferred to subsequent technological operations [58,59].

**Figure 2:**
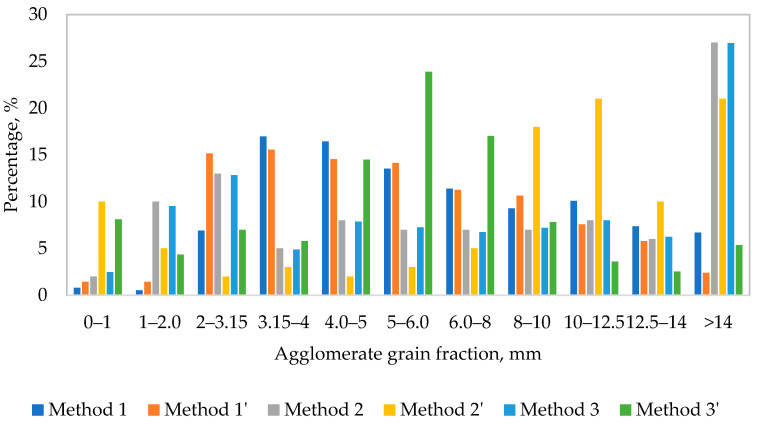
Granulate grain-size (Methods 1, 2, 3; two examples per each method).

Figure 2 shows a comparison of optimum grain-size compositions of agglomerates produced in line with the methods described above (Methods 1–3; two examples per each method). The developed methods show it is possible to produce agglomerates on the basis of waste shavings with varying grain-size composition (with varying granules diameter in relation to the given intended purpose). The best quality granules were obtained from shavings previously mixed with a water glass solution (Methods 2 and 3).

### 3.4. Seed Coatings Based on Collagen Preparations

In view of the nutrition aspect of the plant germination and growth stage, the determining parameter of the used collagen preparations was their amino acid composition. The amino acid analysis (a 6-aminoquinolyl-N-hydroxysuccinimidyl carbamate (AQC) derivatization procedure was applied) indicated the presence of 18 amino acids (aspartate, serine, glutamine, histidine, glycine, arginine, threonine, alanine, proline, cysteine, tyrosine, valine, lysine, methionine, isoleucine, leucine, phenylalanine, and hydroxyproline) in the collagen samples; glycine, alanine, proline, and hydroxyproline were the most abundant amino acids [64,65].

The use of a disc granulator made it possible to form coatings using collagen preparations for seeds of varying dimensions and shapes [61,63]. As a result of the tests, complete, enclosed, spherical, and durable coatings of legumes and rape seeds were formed, increasing their resistance to drought and facilitating plant growth though improving moisture conditions at the germination stage. The length of seedlings for selected seeds and the developed compositions of coatings are shown in Table 5.

An analysis of seedling length showed the effectiveness of collagen preparations in terms of agricultural science as biostimulants [66,67,68], ensuring environmentally friendly crop management, as they enhanced the quality of crops while decreasing chemical inputs. What was also important was their advantage over the model preparation (yellow dextrin and polyvinyl alcohol [69]) and other selected production residues, i.e., solutions of molasses. In addition, the compatibility of the produced coating makes it possible to use a layer of fungicides and zoocides, protecting the seeds against pathogens and pests.

## 4. Discussion

Tanning shavings are a biological material of a diverse, mixed, and heterogeneous nature, with the possible presence of hazardous substances. The risk of using tanning shavings as a renewable raw material should be assessed in terms of the presence of hazardous substances. Nevertheless, tanning shavings can be successfully processed and used in technological and chemical applications. Furthermore, the authors of paper [70] developed a new formula for applications in tanning processes, which reduces volatile organic compounds. Finishing processes are one of the main sources of VOCs in the tanning industry. The volatile organic compounds released by leather materials in different conditions are important for identifying the specific odor of natural leather products [71].

The obtained results show possible wide applications of composite materials produced from tanning shavings. The legitimacy of the proposed solution was also confirmed by the body of literature. There were many examples of solutions for solid waste management in tanneries [61,72,73]. In the building sector, solutions include new acoustic panels based on leather shavings (wet-white) and gypsum, cement, and latex, according to [74]. The research showed sound absorption coefficients of these materials close to those of cork panels or carpets. Moreover, the authors of the paper identified that the calorific values of waste dried hair and leather shavings were found to offer a competitive value in comparison with the biomass products used at present, e.g., wood pellets. Other uses included composite structures made of leather fibers (leather shavings) and cement and leather fibers with rubber powder derived from rubber waste [75]. Leather fibers added to cement mortar panels play an important role in construction due to both their mechanical strength and insulation properties. Waste was introduced as a filler, a powder, or as aggregates in concrete mix, with economic, energy, and environmental benefits [75]. The relevant body of literature gives examples of using leather waste in composite and biocomposite materials that are highly biocompatible, with excellent mechanical properties: biocomposite layers of silica from coatings of silica sols mixed with protein hydrolysate in water/dioxane [76], collagen hydrolysate used as a wall material with chitosan in microencapsulation [77], and other composites [78,79,80,81,82,83]. Further examples include wrapping and packaging materials, such as hydrogels produced with protein hydrolysate with dialdehyde starch, applicable as biodegradable packaging materials for pharmaceutical products, food, and cosmetics [84], paperboard consisting of mixed newspapers and newspapers and leather waste of long fibers [1], paper sheets (with improved properties: water resistance, air permeability, and thermal stability) with multistage disintegration leather shavings [85].

About 60% of industrial chemical products are produced in a granular form [86,87,88,89]. The agglomerate obtained from disc granulation is mechanically stable, durable, as well as easy to transport and store. Granulated materials produced using the developed methods (especially in line with method 1, 1′, 3′) contained a large share of 2–6 mm fraction grains that have the highest application potential in terms of their use in other processing applications (e.g., composite materials).

The strength parameters of the granules were strongly influenced by the application of a water glass solution. This dependence was also obtained in a paper concerning the disc granulation of fly ash and a few percent addition of a filler (bentonite) [90]. Another study investigated the possibility of using a sodium silicate solution in a drum granulation process for biowaste granules [91]. Lower values (compared to tanning shaving granules) of the average destroying force were obtained for carbonation lime mud granulates in which a water solution of molasses was used [55]. The potential application area of the produced granulate involves the production of composite materials based on comminuted collagen fibers, agricultural technology, and as fillers/additives in construction and road construction.

The developed methods of seed pelleting make it possible to produce multicomponent and multilayer coatings with a wide spectrum of activity. Equally important are the type and amount of additives used as well as the sequence of layers. Studies [92,93,94,95] indicated that the growth and primary functioning of plants can be improved by coating seeds with waste-derived materials. Plants can also take up and absorb these amino acids, which sometimes constitute better nitrogen sources than synthetic fertilizers. Furthermore, microelements chelated with hydrolyzed proteins are more easily absorbed by both plant and animal organisms. The literature mentions examples of the use of collagen or collagen and keratin (recovered from the leather industry by-products) preparations as biofertilizers and foliar fertilizers [96,97]. It is possible to use materials recovered from leather waste in cosmetics (e.g., creams), biomedical products (e.g., burn dressings and implant coverings), dietary supplements, and veterinary and feed proteins.

The process of removing chromium from leather waste is based on its contact with solutions of salts, with the addition of acids, and/or anhydrides of acids (acid hydrolysis or alkaline agents, alkaline hydrolysis). There are also combined methods, such as enzymatic and acid hydrolysis. The hydrolysis of leather shavings resulted in a liquid and solid substance with potential applications [98]. The chemical and biochemical method confirmed that the efficiency of protein extraction was 60% and 80%, respectively [99]. Collagen-rich waste can be recycled using methods based on microorganism and enzymatic treatment [100]. There are many dechroming methods that are environmentally friendly and extract native collagen with high molecular weight and yield from chromium-containing leather waste [101]. The economic aspect is also important in the developed methods.

Work is currently underway at the institute in terms of using tanning shavings as a filler in materials intended for the prefabrication of building partitions, agglomerated using secondary raw materials, including waste wood and the textile industry waste. As a result of adding shavings (in the amount of 10%, 20%, and 30%) in wood-like boards pressed under pressure and at a temperature (180 °C), microbiological resistance of the new materials was obtained (in relation to boards without the filler).

## 5. Conclusions

The test of the shavings composition proves their potential reuse in multiple applications in a closed cycle. Leather waste can be processed at an industrial scale to produce value-added products as well as through operations and unit processes (especially those dedicated for the construction, packaging, and agricultural sectors).

The solutions listed above are the confirmed possibilities of using tanning waste in the building sector in accordance with the circular economy model. The properties of the obtained composites can be individually shaped according to the demand and application, through the selection of process parameters. The application of unit processes (such as sieving, granulation, and mixing) in terms of using and processing leather waste (animal-derived biomass) makes it possible to develop new environmentally friendly products that can be composted and that are nontoxic to the ecosystem.

The developed and tested method of waste tanning shavings disc granulation may solve the problem of their processing and make it possible to obtain a durable, mechanically stable, and easy to transport and store semi-finished product. Processing solid leather waste into new raw materials increases the life cycle of the material and reduces its negative environmental impact. Furthermore, considering the limited natural resources, efforts aimed at recovering valuable components from waste materials, while optimizing costs, should be intensified.

## 6. Patents

There are patents and applications resulting from the work reported in this manuscript:

- Method for producing agglomerate from the tanning shavings, Application number P.425268, Exclusive right number Pat.236818
- Method for producing agglomerate from the tanning shavings, Application number P.425277, Exclusive right number Pat.236819
- Method for producing agglomerate from the tanning shavings, Application number P.425287, Exclusive right number Pat.238881
- Method for producing agglomerate from the tanning shavings, Application number P.425288, Exclusive right number Pat.238882
- Method of producing an agglomerate from waste tanning shavings, Application number (P.431100, P.431101, P.431099, P.431102)

## Figures and Tables

**Table 1:** Zingg’s shavings shape classification.

Shape	Volume Fraction [%]
disc-shaped	4.93 ± 0.71
spherical	70.14 ± 1.02
bladed	1.17 ± 0.48
rod-like	23.77 ± 1.52

**Table 2:** Chemical composition of shavings—content of elements in a sample (mg/kg), ND—below the limit of detection.

Element	Concentration (mg/kg)
Ag	ND
Al	47.751
As	ND
Ba	1.697
Bi	ND
Ca	2056.97
Cd	ND
Co	ND
Cr	10,371.6
Cu	ND
Fe	490.561
Ge	ND
Hg	ND
Mg	1380.45
Mn	3.095
Mo	ND
Ni	4.687
Pb	ND
Sb	ND
Se	ND
Sn	ND
Sr	1.193
Ti	ND
V	ND
Zn	6.173
Zr	ND
S	3231.75
P	85.806

**Table 3:** Results of the VOC analysis.

Substance Name	Percentage (%)
3,3-dichloropropine	1.37
methylhydrazine	0.29
formic acid	2.11
methylphosphine/formic acid	2.78
propylene glycol	0.84
benzaldehyde	1.04
carbitol	0.38
benzosulfonosal	8.75
8-methylnonanoic acid	0.32
4-chloro-m-cresol	16.11
2-undecenal	0.29
o-hydroxybifenyl	43.57
tridecanal	0.39
2-(metylotio)-benzotiazol	0.86
2-octylfuran	0.23
triethylene glycol monododecyl ether	0.30
tetradecanal	0.22
myristic acid	0.37
hexadecanal	2.41
pentadecanal	0.26
palmitic acid methyl ester	0.88
palitol acid	0.35
pentadecanoic acid	3.09
linoleic acid methyl ester	0.41
methyl oleate	7.91
9-octadecenoic acid methyl ester	2.51
methyl stearate	0.41
cis-10-heptedecenoic acid	0.63
other substances	0.92

**Table 4:** Value of the Young’s moduli and density of the produced composite materials (average values).

Parameters			Adhesive Medium	
	Homopolymer	Gelatin Glue	Low-Ammonia Natural Latex Glue	Epoxy Resin with a Hardening Agent
Young′s modulus (GPa)	0.0517 ± 0.00981	0.0365 ± 0.00771	0.000594 ± 0.000136	0.000586 ± 0.0000926
Density (g/cm^3^)	0.901 ± 0.08	0.699 ± 0.09	0.509 ± 0.07	0.420 ± 0.08

**Table 5:** Average seedling length for seeds coated with a different composition.

Composition of Coat	Seeds	Average Seedling Length for a Coated Seed, mm
collagen hydrolysate, soot	pea	35.58 ± 1.55 after 10 days following sowing
solutions of molasses, soot	pea	33.28 ± 1.89 after 10 days following sowing
yellow dextrin and polyvinyl alcohol, soot	pea	34.46 ± 0.88 after 10 days following sowing
collagen hydrolysate	rape	12.1 ± 1.51 after 7 days following sowing
yellow dextrin and polyvinyl alcohol	rape	11.56 ± 1.66 after 7 days following sowing
collagen hydrolysate, dolomite	rape	11.45 ± 1.15 after 7 days following sowing
yellow dextrin and polyvinyl alcohol, dolomite	rape	11.075 ± 1.25 after 7 days following sowing
collagen hydrolysate, dolomite, peat	rape	11.75 ± 0.35 after 7 days following sowing
yellow dextrin and polyvinyl alcohol, dolomite, peat	rape	10.7 ± 0.54 after 7 days following sowing

## Data Availability

Not applicable.
